# The ghost of the great imitator: prognostic factors for poor outcome in syphilitic uveitis

**DOI:** 10.1186/s12348-019-0169-8

**Published:** 2019-01-18

**Authors:** Rafael de Pinho Queiroz, Daniele Viana Inês, Felipe Telöken Diligenti, Victor Haygert Schnor, Jacobo Melamed, Wesley Ribeiro Campos, Daniel Vítor Vasconcelos-Santos

**Affiliations:** 1 Departamento de Oftalmologia e Otorrinolaringologia, Faculdade de Medicina da Universidade Federal de Minas Gerais, Av. Alfredo Balena 190. Sala 199, Belo Horizonte, MG 30.130-100 Brazil; 2 Hospital São Geraldo/Hospital das Clínicas da Universidade Federal de Minas Gerais, Belo Horizonte, Brazil; 3 Hospital de Clínicas de Porto Alegre – Universidade Federal do Rio Grande do Sul, Porto Alegre, Brazil; 4 Programa de Pós-graduação em Ciências da Saúde – Infectologia e Medicina Tropical – UFMG, Belo Horizonte, Brazil

**Keywords:** Epidemiology, Prognosis, Sexually transmitted diseases, Syphilis, Uveitis

## Abstract

**Background:**

Syphilitic uveitis is reemerging globally, may lead to any type of intraocular inflammation, and is potentially sight-threatening. We aim to characterize clinical features and prognostic factors in patients with syphilitic posterior uveitis.

**Methods:**

Retrospective chart review at two tertiary university-based referral centers in Brazil. Clinical data, laboratory results, and treatment outcomes were analyzed. Statistical analysis was performed using Fisher’s exact test for categorical variables and Mann-Whitney *U* test for continuous variables.

**Results:**

Forty-four patients (81 eyes) were consecutively diagnosed with syphilitic posterior uveitis between March 2011 and April 2013.Thirty-one were male (70.5%) and the mean age was 43.8 years (range 15–81). HIV confection was disclosed in 12 patients (29.3%). The most prevalent finding was vitritis (85.2%), followed by retinal involvement (76.4%) and optic disc abnormalities (63.5%). After treatment, mean visual acuity improved from 1.2 (20/320) to 0.6 (20/80; median 20/30), but 19 eyes (23.5%) persisted with ≤ 1.0 (20/200). Factors associated with final visual acuity ≤ 1.0 despite therapy were prior use of systemic corticosteroids (*p* = 0.001), higher Venereal Disease Research Laboratory titers (*p* = 0.004), longer duration of symptoms (*p* = 0.024), and worse initial VA (*p* < 0.001).

**Conclusions:**

Syphilitic uveitis is reemerging. Delayed diagnosis and inadvertent use of systemic corticosteroids are potentially modifiable prognostic factors to be considered for possibly improving outcomes.

## Background

Syphilis is a multisystemic, potentially sight-threatening disease caused by the spirochete *Treponema pallidum*. With the advent of penicillin in the middle of last century, global prevalence of syphilis progressively dropped, with parallel decrease in disease morbidity to the central nervous system [1]. However, the incidence of syphilis has now been increasing for more than 15 years, particularly in association with human immunodeficiency virus (HIV) coinfection and with contemporary changes in sexual practices [2–6].

Syphilis may lead to any type of intraocular inflammation, with distinct forms of syphilitic retinitis being characterized in the last decade [7–9]. However, due to the lack of pathognomonic signs and to the fact that uveitis may be the first (and even sole) manifestation of the disease, a high index of suspicion is essential to allow for prompt and appropriate serologic testing, arriving at a definite diagnosis [10].

The prognosis of syphilitic uveitis is classically regarded as favorable, with good response to adequate antibiotic therapy [3, 11, 12]. Few studies tried to characterize factors possibly associated with worse visual outcome in syphilitic uveitis. These reports, however, included relatively small number of patients over long periods of time (up to 30 years) [13–16].

In the context of the reemergence of this old disease and in parallel to other smaller reports [10, 11, 13, 14, 17–19], we aim to investigate clinical features and potential demographic and clinical prognostic factors in a relatively larger series of patients diagnosed with syphilitic posterior uveitis during 2 years at two university-based referral centers in Brazil.

## Methods

After institutional board review and in accordance with the tenets of the Declaration of Helsinki, we retrospectively reviewed charts of patients consecutively diagnosed with posterior syphilitic uveitis from March 2011 to April 2013 at two university-based uveitis referral centers in Brazil. Venereal Disease Research Laboratory (VDRL) and treponema pallidum hemagglutination (TPHA) or fluorescent treponemal antibody absorption (FTA-Abs) assays were routinely performed for all patients. All cases presenting with active posterior uveitis, positive treponemal test results, and favorable response to intravenous penicillin were included, provided that other etiologies were ruled out after appropriate investigations.

Data were collected on age, sex, race, duration of ocular symptoms, affected eye(s), prior use of systemic corticosteroids, best-corrected visual acuity (BCVA), serologic results, HIV coinfection (including CD4 count), cerebral spinal fluid (CSF) analysis, systemic findings, slit-lamp and fundus examination, treatment, and follow-up. BCVA was directly assessed using Early Treatment of Diabetic Retinopathy Study (ETDRS) chart in one center and conventional Snellen chart in the other center, the latter with subsequent conversion of BCVA to LogMAR equivalent.

Statistical analysis was performed using SPSS software version 19 (SPPS, Inc., Chicago, IL, USA) comparing subgroups of eyes attaining final BCVA ≤ 1.0 (20/200) or > 1.0 (20/200). In bilateral cases, the eye with the worst final BCVA was considered for calculation purposes. Categorical variables were compared using Fisher’s exact test, and continuous variables were compared using Mann-Whitney *U* test. A *p* value of < 0.05 was considered statistically significant.

## Results

We included forty-four patients (81 eyes) diagnosed with active syphilitic posterior uveitis between March 2011 and April 2013. Twenty-three (52.3%) were seen at the Uveitis Unit of Hospital São Geraldo/Hospital das Clínicas da Universidade Federal de Minas Gerais between April 2011 and April 2013 and 21 (47.7%) at the Uveitis Unit of Hospital de Clínicas da Universidade Federal do Rio Grande do Sul, between March 2011 and February 2013. The majority of patients were male (70.5%), with a mean age of 43.8 years (range 15–81). Intraocular inflammation was bilateral in 37 patients (84.1%) and unilateral in 7 (15.9%).

At presentation, 11 patients (25.0%) displayed cutaneous lesions suggestive of secondary syphilis and one (2.3%) had genital lesions. All, except three patients, had a positive VDRL, with titers ranging from 1:4 to 1:4096 (median 1/64). Treponemal test results could not be retrieved for five patients, but these all had high VDRL titers (ranging from 1:64 to 1:256).

Duration of ocular symptoms before presentation ranged from 4 to 720 days (mean 104.5 days; median 38.5 days). Nine patients (20.5%) had inadvertently received systemic corticosteroids (oral or parenteral) prior to the definite diagnosis. Twelve patients (29.3%) were HIV-positive, and three patients had unknown HIV status. CD4 count was available for nine patients, with a mean of 260.1 cells/mm^3^ (range 50–569).

Lumbar puncture (LP) was performed in 33 patients (75%), with 23 (69.7%) showing CSF changes such as positive VDRL, pleocytosis, and/or hyperproteinemia. Among HIV-coinfected patients, 10 underwent LP, of which nine (90%) had CSF abnormalities, in contrast to CSF positivity of 61.9% among those HIV-negative.

BCVA at presentation ranged from 0.0 (20/20) to no light perception [mean 1.2 (20/320); median 1.0 (20/200)]. Slit lamp examination and fundus examination showed vitritis in 69 eyes (85.2%). Fundus examination was precluded due to dense vitritis in three eyes of two patients, and these were excluded from the analysis of optic disc and retinal, choroidal, and retinal vascular lesions. Initial B-scan, however, showed attached retinas in all of them. Ocular features, including initial and final BCVA, fundus examination at presentation, and complications, are summarized in Table 1. Of note, 11 eyes (13.6%) of eight patients (18.2%) presented with features of acute syphilitic posterior placoid chorioretinitis.

**Table 1:** Ocular features and complications in 81 eyes of 44 patients with syphilitic posterior uveitis

	*N* (%)
BCVA at presentation	
NLP	4/81 (5.0%)
LP–20/200	44/81 (54.3%)
20/160–20/63	10/81 (12.3%)
20/60–20/20	23/81 (28.4%)
Final BCVA	
NLP	4/81 (5.0%)
LP–20/200	15/81 (18.5%)
20/160–20/63	8/81 (9.8%)
20/60–20/20	54/81 (66.7%)
Fundus examination	
Vitritis	69/81 (85.2%)
Optic disc abnormalities	47/74 (63.5%)
Retinal involvement	60/78 (76.9%)
Retinal vasculitis	36/78 (46.2%)
Necrotizing retinitis	17/78 (21.8%)
Posterior placoid chorioretinitis	11/77 (14.3%)
Non-necrotizing retinitis	11/78 (14.1%)
Punctate inner retinal lesions	6/78 (7.7%)
Others	9/78 (11.5%)
Ocular complications	
Cataract	8/81 (9.9%)
Epiretinal membrane	4/81 (4.9%)
Retinal detachment	10/81 (12.3%)
Optic atrophy	4/81 (4.9%)
Others	14/81 (17.3%)

*BCVA* best-corrected visual acuity, *NLP* no light perception, *LP* light perception

All patients were treated with intravenous benzylpenicillin 4 million IU q4h for 12–21 days (mean 15.1; median 14 days), except one patient, who initially presented with posterior placoid chorioretinitis at our emergency care unit, documented by color fundus photography, but by the time of the consultation at the Uveitis Unit, intraocular inflammation had spontaneously resolved. He was then treated with three doses of benzathine penicillin 2.4 M units. A tapering regimen of systemic corticosteroid was also prescribed to 33 patients (75%).

Intraocular inflammation resolved after therapy in all patients (mean 8.3 months, median 7 months, range 1–24 months). Final BCVA ranged from 0.0 (20/20) to no light perception [mean 0.6 (20/80); median 0.2 (20/30)]. Nineteen eyes (23.5%) had BCVA ≤ 1.0 (20/200) at last follow-up visit, and 54 eyes (66.7%) had final BCVA of ≥ 0.5 (20/60). Of the 37 bilateral cases, six (16.2%) ended up legally blind, with a final BCVA ≤ 1.0 (20/200) in the better eye. Ocular complications developed in 40 eyes (49.4%) of 31 patients (70.4%), with the most prevalent being retinal detachment (RD) in 10 eyes (Table 1**)**.

Final BCVA ≤ 1.0 (20/200) was statistically associated with longer duration of symptoms before diagnosis (*p* = 0.024), prior inadvertent use of systemic corticosteroids (*p* = 0.001), higher VDRL titers (*p* = 0.004), and worse initial BCVA (*p* < 0.0001), but not with sex (*p* = 0.131) or HIV coinfection (*p* = 0.300). Older age and presence of necrotizing retinochoroiditis were shown not to be statistically significant, but might be related to poor outcomes—Table 2.

**Table 2:** Prognostic factors associated with syphilitic posterior uveitis in 44 patients

	Final BCVA < 1.0 (20/200)	Final BCVA > 1.0 (20/200)	*p* value
Sex			
Male	11 (84.6)	20 (64.5)	0.282*
Female	2 (15.4)	11 (35.5)	
Age (mean per eye)	51.7 ± 18.3	40.5 ± 15.9	0.070**
HIV coinfection			
Positive	5 (38.5)	7 (25.0)	0.469*
Negative	8 (61.5)	21 (75.0)	
Median VDRL titer	1/512 [0–4096]	1/64 [0–4096]	0.004**
Prior inadvertent use of systemic steroids			
Yes	7 (53.8)	2 (6.4)	0.001*
No	6 (46.2)	29 (93.6)	
Presence of necrotizing retinochoroiditis			
Yes	6 (46.2)	5 (16.1)	0.057*
No	7 (53.8)	26 (83.9)	
Mean time of symptoms before diagnosis (days)	176.8 ± 250.2	74.8 ± 133.1	0.024**
Mean initial BCVA (LogMAR)	2.1 ± 0.6	1.1 ± 0.7	< 0.001**

Continuous variables are expressed as median [range] or mean ± standard deviation

Categorical variables are expressed as absolute number (%)

*BCVA* Best-corrected visual acuity

*Fisher’s exact test

**Mann-Whitney *U* test

In addition, inadvertent use of systemic corticosteroids and VDRL titers seem not to be correlated, when comparing the group of patients who received and did not receive corticosteroids prior to the diagnosis (*p* = 0.56). Furthermore, high VDRL titers (≥ 1/128) were associated with poor outcome [odds ratio 10.86 (CI 2.2–83.9)], as well as use of systemic corticosteroids with no antibiotic coverage [odds ratio 15.43 (CI 2.7–131.2)].

## Discussion

After a steady decline during the 1990s, the global incidence of syphilis is again increasing [20], with ocular manifestations being on the rise, especially among men [2–6]. Posterior uveitis is the most common ocular manifestation [12, 21, 22], regardless of HIV status. Herein, we present data from 44 patients with syphilitic posterior uveitis consecutively seen at two referral centers in Brazil over a period of 2 years (average of 22 patients/year), in contrast to other published series collecting cases over much longer periods of time, with the exception of the study by Mathew et al. [11] **(**Table 3). In addition to relevant demographic and clinical data, we also identified potentially modifiable prognostic factors, helping to raise awareness of this ancient, but intriguing disease. Agreeing with the literature [10, 11, 13–15, 17–19, 21–23], uveitis occurred more frequently in middle-aged men, being bilateral in most patients (84.1%). HIV coinfection was present in nearly one third of the patients, highlighting the importance of HIV testing in individuals with syphilis. Also in line with other reports [14, 21, 22], CSF changes consistent with neurosyphilis were more common in HIV-positive patients than in immunocompetent ones.

**Table 3:** Comparison of published series on syphilitic uveitis

Reference	Country	Number of centers	Number of patients	Period (no. of years)	No. of patients/year†
Present series	Brazil	2	44	2011–2013 (2)	22
Hoogewoud et al. [24]	France	2	66	2003–2016 (14)	4.7
Bollemeijer et al. [15]	Netherlands	5	85	1984–2013 (29)	2.9
Tsuboi et al. [27]	Japan	1	20	1997–2015 (18)	1.1
Zhang et al. [16]	China	1	15	2012–2015 (3)	5
Shen et al. [28]	China	1	13	2009–2014 (5)	2.6
Carbonnière et al. [29]	France	1	27	2000–2013 (13)	2.1
Lee et al. [30]	USA	1	16	2008–2014 (6)	2.7
Moradi et al. [19]	USA	1	35	1984–2014 (20)	1.75
Northey et al. [17]	Australia	1	25	2007–2012 (5)	5
Yap et al. [31]	Singapore	1	12	2004–2009 (5)	2.4
Fonollosa et al. [14]	Spain	8	50	2000–2012 (12)	4.2
Mathew et al. [11]	United Kingdom	National	41	2009–2011 (2)	20.5
Yang et al. [10]	China	1	19	2004–2011 (7)	2.7
Li et al. [32]	USA	1	13	1991–2009 (18)	0.7
Balaskas et al. [13]	Switzerland	1	26	1999–2009 (10)	2.6
Fonollosa et al. [5]	Spain	2	12	2005–2007 (2)	6
Anshu et al. [6]	Singapore	1	22	1995–2006 (11)	2
Parc et al. [33]	France	1	10	2001–2004 (3)	3.3
Doris [2]	England	1	6	2004 (1)	6
Chao et al. [3]	USA	1	4	2005 (1)	4
Tran et al. [34]	France	1	12	2001–2003 (2)	6
Ormerod et al. [35]	USA	1	21	1990–1993 (3)	7
Browning et al. [18]	USA	1	14	1986–1999 (13)	1.1
Villanueva et al. [36]	USA	1	20	1993–1996 (3)	6.7
Shalaby et al. [37]	USA	1	13	1983–1995 (12)	1.1
Barile and Flynn [38]	USA	1	24	1989–1994 (5)	4.8
Tamesis and Foster [39]	USA	1	25	1983–1989 (6)	4.2
Schlaegel and Rao [40]	USA	1	28	1970–1980 (10)	2.8

† Total number of patients included in each study divided by the study period in years

Our patients were managed with intravenous benzylpenicilin and adjunctive corticosteroids. Visual prognosis following treatment was good, with the majority reaching final BCVA ≥ 0.5 (20/60). A significant proportion of our patients, however, persisted with decreased BCVA due to ocular complications, mainly RD and optic atrophy. Factors associated with a poorer prognosis (BCVA ≤ 1.0) included prior inadvertent use of systemic steroids, delayed diagnosis, higher VDRL titers, and worse initial BCVA. Some of these associations have been suggested in earlier reports [14–16, 19, 24] and highlight the importance of early recognition of this entity, invariably performing adequate serological tests for syphilis in all patients presenting with uveitis, for prompt institution of adequate treatment. Interestingly, our finding of higher VDRL titers being associated with worse prognosis may be related to increased treponemal load and disease activity [25]. HIV coinfection was not associated with a worse visual prognosis, similarly to other studies [11, 14, 15, 17, 19]. A recent report from China, however, has found this association [10].

A prospective study from the UK published by Mathew et al. [11] with a comparable number of patients over a similar time span reports different results and final outcomes, when compared to the present series. The overall better prognosis in the British paper might be related to an earlier diagnosis at the early phases of secondary syphilis. This is highlighted by the shorter duration of symptoms before diagnosis, better mean VA at presentation, and the fact that the study looked at ocular presentation in early syphilis. Our series includes a larger number of bilateral cases and some patients with low and a few with even negative VDRL titers, indicating that syphilis might have been present and undiagnosed in our patients for a longer period of time. This late presentation, in addition to prior use of corticosteroids and the exclusion of subjects with anterior uveitis only, might explain, at least in part, our worse outcome when compared to Mathew et al. [11].

Recently, a large retrospective study analyzed prognostic factors for syphilitic uveitis [24] as predictors of ophthalmological recovery 1 month after treatment onset. The authors could find that baseline BCVA and initial improvement at 1 week were associated with recovery at 1 month and periocular dexamethasone injections and methylprednisolone pulses negatively affected the outcomes. However, complications might still arise after 1 month of treatment onset, as previously reported [26].

Our study is limited by its retrospective design and relatively short follow-up. On the other hand, the concentration of cases over a relatively short period of time (average of 22 patients/year), when compared to previous reports (Table 3), is to be considered.

In conclusion, syphilitic uveitis is reemerging globally. Delayed diagnosis and inadvertent use of systemic corticosteroids are potentially modifiable prognostic factors to be considered for possibly improving outcomes. Proper recognition of its forms of presentation, prognostic factors, and complications may be helpful for better diagnosis/management of this fascinating disorder.

## Abbreviations

BCVA: Best-corrected visual acuity
CSF: Cerebral spinal fluid
ETDRS: Early Treatment of Diabetic Retinopathy Study
FTA-Abs: Fluorescent treponemal antibody absorption
HIV: Human immunodeficiency virus
RD: Retinal detachment
TPHA: Treponema pallidum hemagglutination assay
VDRL: Venereal Disease Research Laboratory
